# Relationship between speed perception and eye movement—A case study of crash-involved and crash-not-involved drivers in China

**DOI:** 10.1371/journal.pone.0229650

**Published:** 2020-03-11

**Authors:** Fuwei Wu, Rui Fu, Yong Ma, Chang Wang, Zhi Zhang

**Affiliations:** 1 Key Laboratory of Transportation Industry of Automotive Transportation Safety Enhancement Technology (Chang’an University), Xi'an, China; 2 School of Automobile, Chang'an University, Xi'an, China; Tsinghua University, CHINA

## Abstract

Speed perception tests are already used in several countries as part of the driver licensing curriculum; however, this test is not compulsively required in China. The purpose of this study was to investigate the relationship between speed perception and eye movement for different driver groups. Forty-eight drivers, including 28 crash-involved (CI), with rear-end or side collisions, and 20 crash-not-involved (CNI) drivers, were recruited for the speed perception experiments. Drivers’ reaction characteristics as well as eye movement data were analyzed. The results showed that CI drivers were more likely to overestimate the speed of visual stimuli and react in advance. The speed perception of CI drivers was more accurate than that of CNI drivers for visual stimuli with middle to high moving speeds, indicating that CNI drivers are more cautious and conservative when driving. Regarding eye movement, significant differences in saccade speed were found between the CI and CNI drivers in the occlusion area under high speed and the occlusion ratio. The relationship between visual pattern and speed perception accuracy was found to some extent. Implications of the speed perception test for the driver aptitude test were discussed.

## 1. Introduction

Speed perception refers to the ability of people to estimate the speed of objects and is one of the prerequisites for safe driving in human factors engineering. Drivers’ speed perception is composed of the perceived speed of their own vehicle and that of other objects. Generally, drivers perceive the exact speed of their own vehicles mainly through the speedometer, but doing so causes their eyes to turn away from the road and increases the risk of accidents. Hence, many drivers usually estimate the speed of their own vehicle according to the traffic environment. It is believed that misperception of the speed of their own vehicles is likely to cause speeding accidents. In addition, drivers have to judge the speed of other traffic participants all the time and take appropriate actions to avoid potential collisions. Speed perception is related to the human visual system. Given that animals with foveated eyes use two different types of eye movements to keep their gaze near a moving target, namely, high-speed, discrete saccades and low-speed, continuous smooth pursuit [1], speed perception is a combination of both saccade and pursuit eye movement behaviors.

There have been several studies on speed perception. In the study of Wu et al. [2], participants were asked to judge the speeds of a vehicle from the driver’s perspective according to video clips selected from naturalistic driving recordings. The results showed that speeds were most accurately estimated in the 25–35 mph range, but speeds below this range tended to be overestimated, while speeds above this range were more likely to be underestimated. Regarding the image sizes of the video clips, the actual field of view in the real world, a significant effect of image size on estimation error was found (F_3, 78_ = 45.5, p<0.001). However, no effect of the image size on driving speed perception was found in their early study [3].

The role of vision and light flow in speed perception was first proposed by Gibson [4]. The optic angle denotes the angle that is formed between a line to the focal-point-of-view and a line connecting the stimulus with the counter image projected on the retina of the driver’s eye. Lee [5] mathematically translated the optic flow as the temporal differentiation of this optic angle. Moreover, visual feedback regarding self-motion is provided by the optic flow, which can be used to estimate the direction of self-motion (heading) rapidly and efficiently [6]. Based on the optic flow theory, Hirofumi et al [7] proposed a theoretical model regarding driver perception of vehicle speed and found that the model is statistically valid.

Speed perception is affected by visual adaptation. Traffic psychology studies generally believe that speed perception depends on the speed previously given. Adaptation to a given speed regularly leads to a reduction in perceived speed and an improvement in speed recognition ability [8]. Once the driver adapts to a low speed, the perceived speed of a high-speed test will be slightly improved [9]. In particular, when driving at a constant speed for a long time and then slowing down on an expressway off-ramp without paying attention to the speedometer, the actual speed may be higher than the expected speed of the driver [7], which is a very typical illusion of speed in driving.

In addition, speed perception of the driver’s own vehicle is also related to hearing. The psychophysics of speed and motion perception is jointly determined by the auditory, visual and audiovisual cues [10, 11]. Without vehicle noise, drivers' ability to maintain the correct speed is worse than that with vehicle noise, and the difference is greater at high speeds [12]. When the noise reduces, drivers are likely to underestimate the speed of their own vehicles, which might potentially encourage them to drive faster and put them at greater risk of a crash [13].

While driving, it is also important to judge the speed of other traffic participants. Drivers often encounter other traffic participants occluded by buildings, trees or large vehicles. At this time, they need to accurately judge the speed of other traffic participants and take the right operations in a timely manner to reduce the possibility of traffic conflicts. The accuracy of speed perception is affected by many factors. Previous studies have shown that human speed perception is related to contrast [14–16]. Objects with large contrast are easy to recognize, and the corresponding speed judgment is more accurate. For both binocular images moving in depth and monocular images translating laterally, decreased contrast leads to decreased perceptual speed [16, 17]. Both prediction distance and stimulus velocity are significant variables [18]. Observers tend to overestimate the speed of small objects, and an increase in size of moving objects will lead to slower perceived speed or longer visual movement time [19]. In addition, speed perception is affected by flicker and dynamic stripe patterns. The perceived speed of intermittent stimuli depends on the moving speed and flashing frequency of objects. Slow moving speed and high flashing frequency of stimuli lead to overestimation of speed, while fast moving speed and low flashing speed frequency lead to underestimation of speed [20]. Dynamic stripe patterns moving in the same direction as that of the target are found to increase the perceived speed of that target [21, 22]. Reduced illuminance levels are associated with increased speeding [23]. Moreover, visual pursuit is asymmetric, horizontal and downward, which are more important. The speed of tracking downward motion is significantly faster and smoother (less or later rapid scanning) than that of tracking upward motion. Compared with vertical motion, horizontal motion is tracked more accurately and smoothly [24].

Speed perception is widely used in driving ability assessments in several countries, such as the United Kingdom and Austria. If the perceived speed is slower than the actual motion speed of the object, it indicates that the driver is impatient, and the driver is prone to speeding in the driving process [25]. Compared with professional drivers, the perceived speed of drivers with few years of driving experience is relatively high [26], while the average perceived speed of drivers with bad driving behaviors (with violation records) is lower than that of professional drivers.

At present, representative methods for testing drivers' speed perception ability have been formed, such as the Vienna test of traffic psychology in Austria [27], the CRT test in Japan [28], and China's transportation industry standard *Road transport driver–Appraisal way of automobile driving aptitude test* (JT/T442-2014) [29], which all include speed perception test items. The basic principle is to divide the test panel into a visible area and an occlusion area. A visual stimulus signal (such as a red or green ball) enters the occlusion area from the visible area. When the subjects assume the ball reaches the end of the occlusion area, they press the reaction button. Response time error is used to represent the evaluation index of the speed perception ability of the drivers.

Based on the analysis above, the previous literature has mainly been limited to the factors that influence speed perception. There is a need to explore the visual mechanism during speed perception. The research presented here investigates the speed perception characteristics and the visual mechanism of crash-involved and crash-not-involved drivers. Thus, it might provide useful ideas for the speed perception ability evaluation of drivers.

## 2. Methods

The ethics committee of Chang'an University approved this study.

### 2.1 Participants

A total of 48 participants aged 21 to 50 were recruited for the study. All drivers held a C1 or above driver’s license of China and had normal vision (above 5.0). The participants were composed of 28 CI (15 male, 13 female) and 20 CNI (10 male, 10 female) drivers according to whether they had been involved in at least one accident over the past three years. Each of the CI drivers (M = 34.2 years, SD = 9.4) had been involved in at least one rear-end collision or side collision, which might have been caused by misperception of the speed of other vehicles, while the CNI drivers (M = 36.7 years, SD = 10.6) had no accidents in the same period. An independent sample t-test showed that there were no significant differences in age (t = -1.021, p = 0.313), gender (t = 0.239, p = 0.812) or driving experience (t = -0.634, p = 0.529) between the CI and CNI groups.

### 2.2 Apparatus

**Fig 1:**
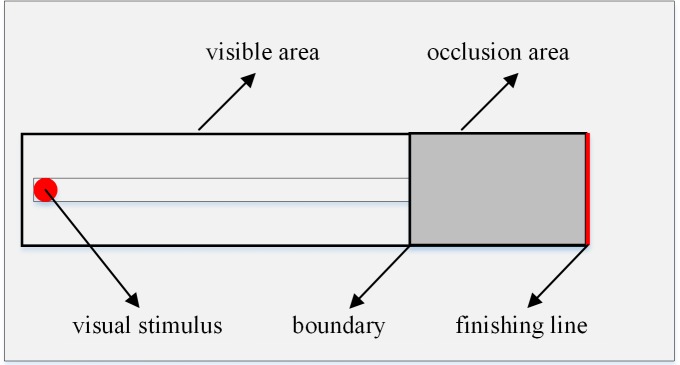


The speed perception program was developed using the Unity 3D platform (Fig 1). The test scene was played on a 60” TFT monitor at a resolution of 4096 × 2160. Participants were seated 120 cm away from the screen with a horizontal visual angle of 45.6° and a vertical visual angle of 32.2°. The test interface was composed of a visible area and an occlusion area. The visual stimulus (a red ball) moved at a preset speed from left to right uniformly. In the visible area, participants could see the moving stimulus. In the occlusion area, participants needed to judge the speed of the stimulus according to their mental perception. The participants were expected to press the reaction button when they assumed the stimulus reached the finishing line, and the reaction time was recorded automatically.

Meanwhile, a noncontact eye tracker (FaceLAB 5.0, SeeingMachine, Australia) was used to collect the eye movement data of the subjects at a sampling frequency of 30 Hz to analyze the visual characteristics during the speed perception process.

### 2.3 Procedure and design

#### 2.3.1 Occlusion ratio

The horizontal visual angle basically falls in the range of (-16.7°, 16.7°). The occlusion ratio represents the proportion of the test scenario that is occluded. There are three occlusion levels: s1 = 20%, s2 = 30%, and s3 = 40%. Hence, the angles of the visible and occlusion boundaries are 3.37°, 6.74° and 10.11°, respectively.

#### 2.3.2 Size of visual stimulus

The size of the visual stimulus is generally measured by the visual angle. In industry criteria JT/T442 of China, the diameter of the stimulus is set to 18 ± 5 mm, and the detection distance is 2.5 m, with a corresponding visual angle of the diameter of approximately 0.4°. In another study, the visual stimulus angle of Sokolov was set to 0.15°. In our study, the diameter of the visual stimulus was set to 18 mm, corresponding to a visual angle of 1°.

#### 2.3.3 Speed of visual stimulus

Under the premise of a certain total length, the smaller the proportion of the occlusion area, the more accurate the subjects' perception of the motion speed of visual stimuli, but the error will increase if the proportion of the occlusion area is too large. It is believed that visual moving speed might affect the perception accuracy. In this paper, the moving speed of the visual stimulus was set to v1 = 2.5°/s, v2 = 5°/s and v3 = 10°/s of the interface length.

#### 2.3.4 Procedure

The experiment was designed with the following sample interval: 2 (driver groups) × 3 (motion speed) × 3 (occlusion ratio). Each level was repeated 3 times, and each subject conducted a total of 27 trials. The subjects then placed their chins on a chin rest to ensure that the visual angle of stimulus observed by different subjects remained the same. All subjects were required to take breaks for approximately 5 minutes. Before the formal test, the eye tracker was adjusted and calibrated properly. To reduce the learning effect, the order of the 27 trials was random. During the experiment, the subjects were expected to press the button when they thought the visual stimulus reached the designated position marked with a red line.

#### 2.3.5 Data analysis

*1*. *Reaction in advance*. The dependent variables include reaction behavior and visual behavior. The reaction behavior was analyzed to determine any reaction in advance, referring to the subjects’ reacting before the visual stimulus reached the finish line, and the reaction accuracy was defined as the time error with regard to the standard amount of time needed for the stimulus to reach the finish line. In this paper, negative reaction time represented a reaction before the stimulus signal reached the end point, while a positive value represented the operation after the stimulus signal reached the end point.

*2*. *Saccade speed*. The eye movement data were sampled at 30 Hz. Visual behavior was analyzed for saccade speed, the number of times each subject looked backward and any visual patterns. A video of a scene overlaid with fixation points was used to determine the starting and ending frames of eye movement data in the visible area. The ending frame of the occlusion area was calculated according to the subjects’ response time and the speed of the visual stimulus. Saccade speed is defined as the average angular velocity of the eye in pursuit of the visual stimulus.

**Fig 2:**
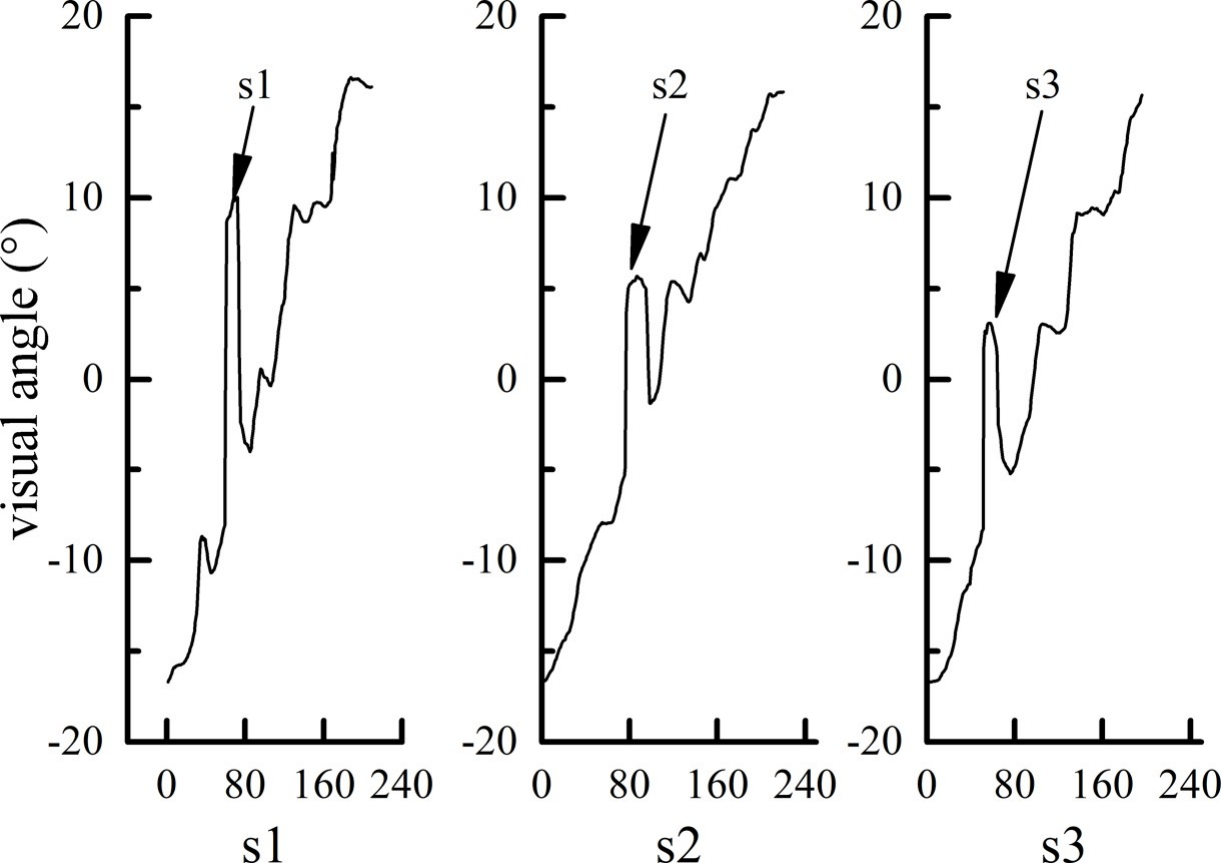


*3*. *Recall times*. Recall refers to the process in which the subject's gaze follows the visual stimulus, with the fixation point jumping to the front of the visual stimulus signal (the boundary of the visible and occlusion areas) and then returning back to the visual stimulus (Fig 2). Recall mainly occurs in the visible area.

We analyzed whether the speed of the visual stimulus and the proportion of the occlusion area had a significant influence on the reaction time. In addition, three-way repeated ANOVA was conducted to analyze the differences in speed perception ability. Data extraction and screening were conducted in MATLAB version 2017 (The MathWorks, Inc.). Statistical analysis, such as the independent sample t-test, was performed in SPSS 25 (IBM Corp.).

## 3. Results

### 3.1 Reaction characteristics

#### 3.1.1 Reaction in advance

**Fig 3:**
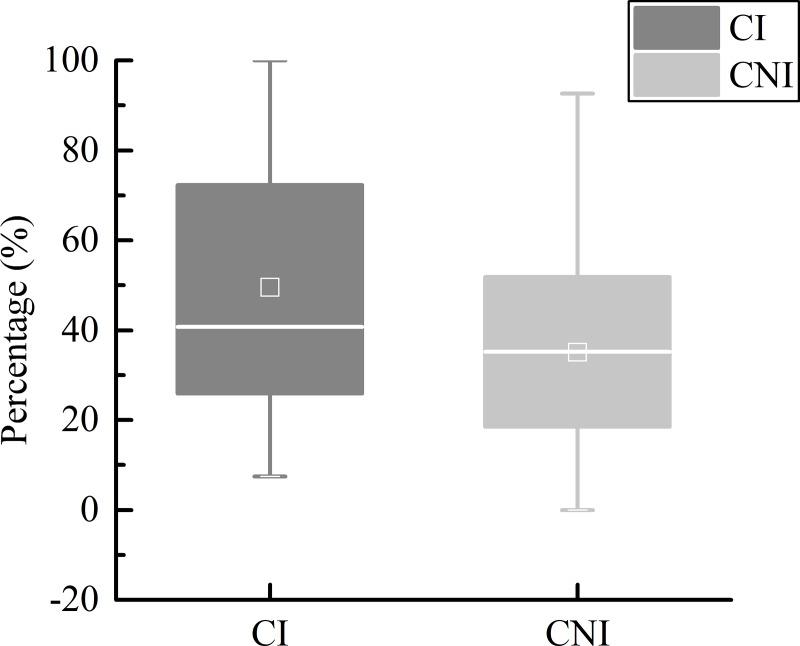


In the experiments, the participants were required to press the button when they assumed the stimulus reached the finish line of the occlusion area. The response time error of the subjects was normally distributed. To some extent, pressing the button before the stimulus reached the end represented an overestimation of the speed of the stimulus, reflecting that the participants had psychological characteristics of impatience. Therefore, the index reaction in advance was proposed. A reaction before the visual stimulus reached the end was marked as 1; otherwise, it was marked as 0. Without considering the speed of the stimulus and the occlusion ratio, statistics were made on the condition of the participants in the CI and CNI drivers’ reaction in advance (Fig 3).

The results showed that the proportion of the CNI drivers that reacted in advance was 35.1% ± 23.5%, while the advance action proportion of the CI drivers was 49.5% ± 29.6%. The results indicated that CI drivers tend to overestimate the moving speed of objects in the process of speed perception.

The odds ratio (OR) was used to calculate the difference in advance action between the two groups. The odds ratio, also known as the cross product ratio, is used to compare the probability of two kinds of events. This indicator reflects how much some kind of conjecture occurs compared with its opposite case. How much more likely it is that some conjecture occurs is reflected in the odds compared with the opposite case.

The probabilities of reaction in advance of CI and CNI drivers were set as *π*_1_ and *π*_2_, respectively. The odds of reaction in advance of CI and CNI drivers are defined as follows:

$$odds_{1}=\frac{\pi_{1}}{1-\pi_{1}}$$ (1)

$$odds_{2}=\frac{\pi_{2}}{1-\pi_{2}}$$ (2)

Then, the odds ratio of reaction in advance of CI over CNI drivers was defined as *θ*.

$$\theta =\frac{odds_{1}}{odds_{2}}=\frac{\pi_{1}(1-\pi_{2})}{\pi_{2}(1-\pi_{1})}$$ (3)

**Fig 4:**
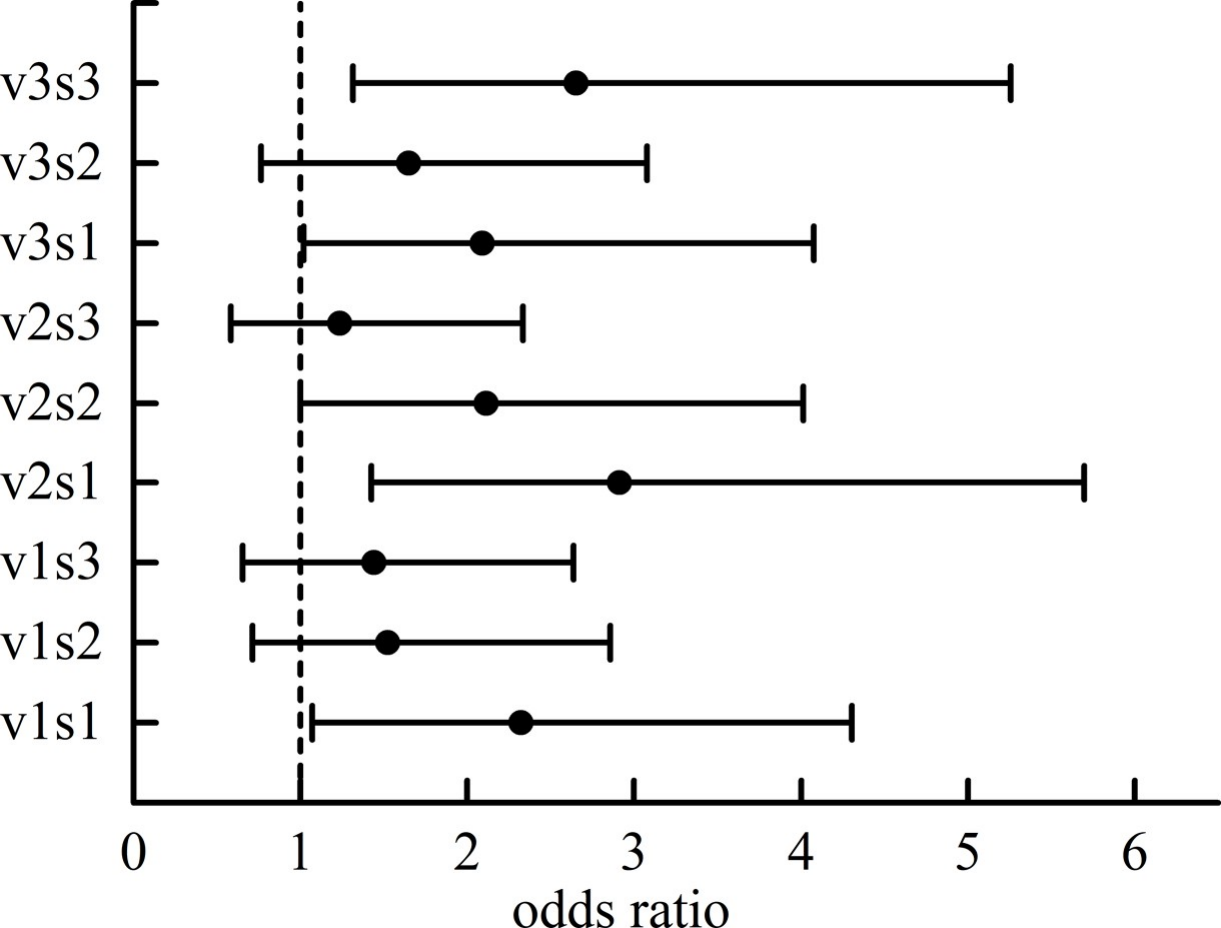


The odds ratios under different speeds of the visual stimulus and different occlusion ratios were calculated in SPSS 25 (Fig 4).

When the odds ratio is greater than 1, the probability of success is greater than the probability of failure. The odds ratios of CI over CNI drivers were greater than 1 under all situations, indicating that crash-involved drivers are more prone to overestimate the speed of visual stimuli than are crash-not-involved drivers. This paper showed evidence that crash-involved drivers had a significantly higher proportion of advanced actions than did crash-not-involved drivers (t(56) = -2.067, p < 0.05). It should be noted that the crash-involved drivers overestimated the speed of the stimulus.

#### 3.1.2 Speed perception accuracy

**Fig 5:**
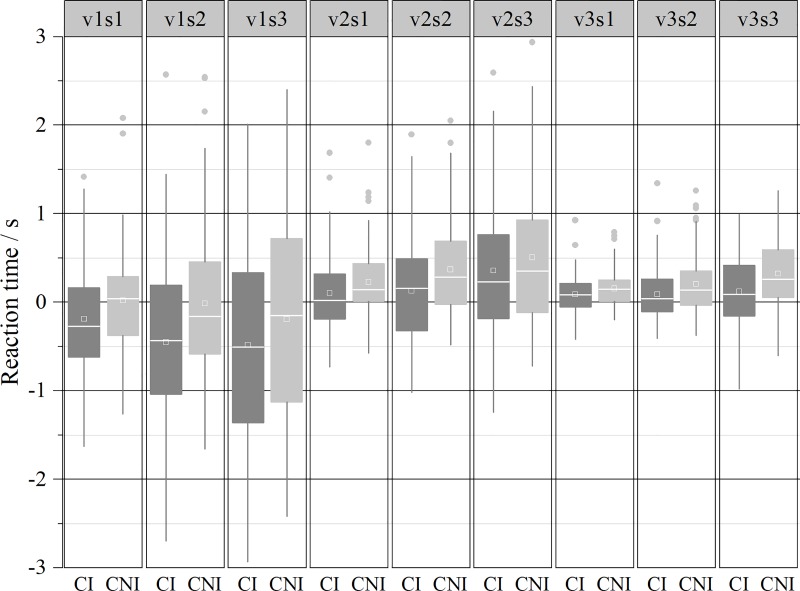


The speed perception accuracy of CI and CNI drivers under different conditions is shown in Fig 5.

The results showed that in either case, the response time of CI drivers was shorter than that of CNI drivers (Fig 5). That is, the CI drivers reacted earlier than the CNI drivers, which is consistent with the previous conclusion of the odds ratios of advance action. Under the v1 situation, the CNI driver response was more accurate, and under v2 and v3, the response of the CI group was more accurate. Generally, the CI drivers conducted operations in advance of the CNI drivers, which indicates that they are impatient and that the CNI drivers are conservative and will not take risks in response to traffic events.

A repeated measures 2 (driver group) × 3 (moving speed) × 3 (occlusion ratio) ANOVA was assessed for systematic trends, but no significant effect was found (F(2.934, 225.926) = 1.020, P = 0.383 > 0.05). A significant interaction was found between driver group × occlusion ratio (F(1.727, 133.002) = 3.751, P = 0.032 < 0.05) and moving speed × occlusion ratio (F(2.870, 220.987) = 12.486, P < 0.001). However, there was no significant interaction between driver group × moving speed (F(1.515, 116.654) = 2.693, P = 0.086). Greenhouse-Geisser corrections (ε) were used where assumptions of sphericity were violated.

Then, the interaction of the driver group and occlusion ratio on speed perception accuracy was examined under different speeds of the visual stimulus. The result showed that there was no statistically significant interaction under the v1 (F(1.741, 134.092) = 1.758, P = 0. 181 > 0.05) and v2 (F(1.892, 145.647) = 2.023, P = 0.139 > 0.05) conditions. A significant interaction (F(1.813, 139.605) = 3.514, P = 0. 037< 0.05) was found under the v3 condition.

### 3.2 Eye movement

After the missing or abnormal eye movement data were excluded, the eye movement data of 23 CI and 13 CNI participants were finally used for further analysis.

#### 3.2.1 Saccade speed

**Fig 6:**
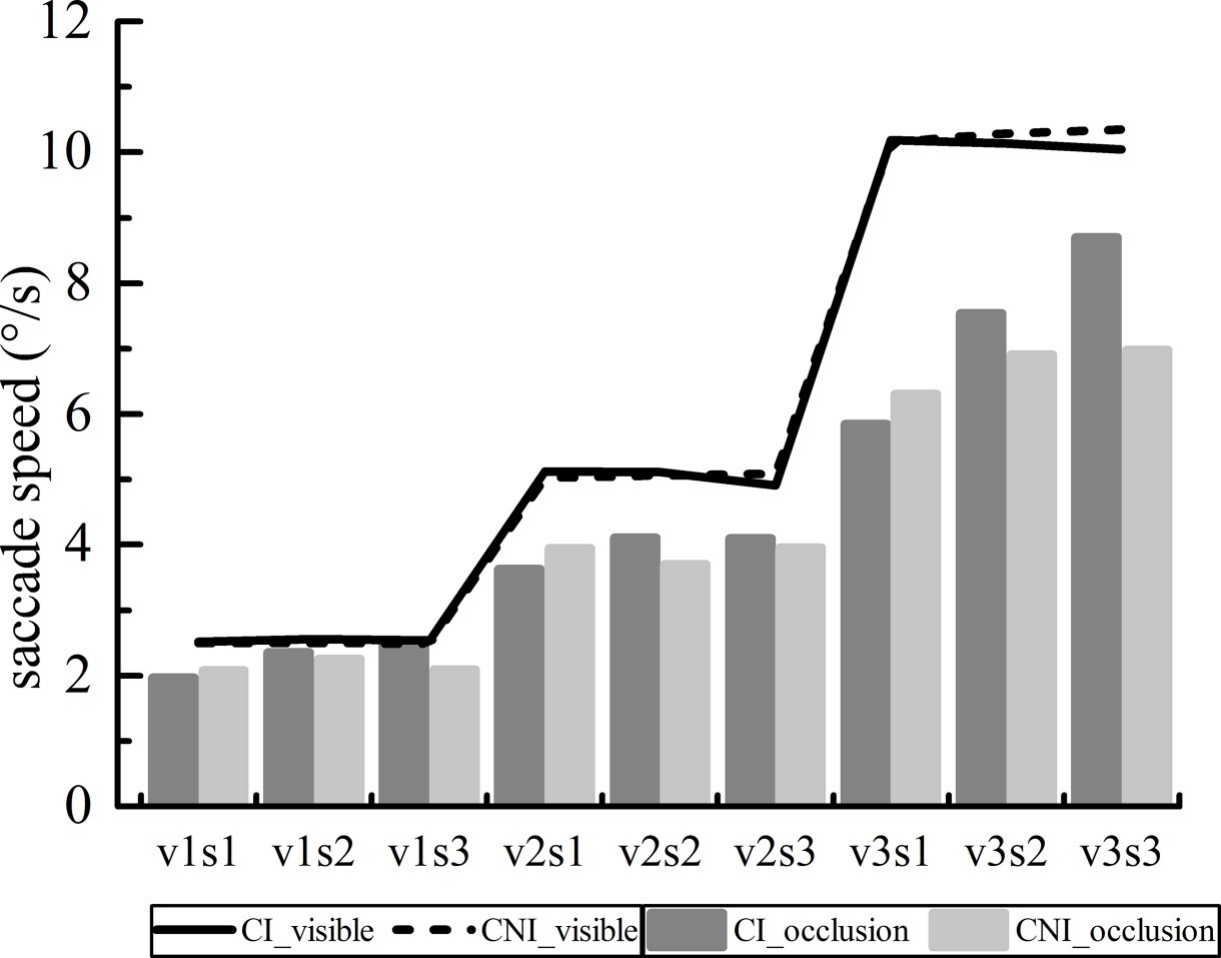


The saccade speeds in the visible and occlusion areas were calculated for different groups of subjects. It is presumed that in the visible area, there should be no difference in the saccade speeds between CI and CNI drivers since the visual stimulus is visible. However, in the occlusion area, there might be a difference between the two groups in terms of the saccade speed. In the occlusion area, drivers differed in psychological estimation patterns, and reaction accuracies varied. Therefore, we assumed that the greater the moving speed of the visual stimulus, the greater the variance in the saccade speeds. The saccade speeds of different subjects in the light and occlusion areas are shown in Fig 6.

It can be seen from Fig 6 that the saccade speed of subjects with different past histories of accidents was basically the same in the visible area, and no significant difference was found under the target speeds of v1 (t = 10.765, p = 0.112), v2 (t = 2.465, p = 0.975) and v3 (t = 1.958, p = 0.454). This result might have occurred because the visual stimulus moves at a constant speed in the visible area, and the subjects were able to follow the visual stimulus with smooth pursuit eye movement. However, when the visual stimulus was hidden in the occlusion area, the participants could not continue to perform the pursuit eye movement and would only make decisions via psychological estimation.

The maximum difference in saccade speed in the visible area was only 3.44% (under the v2s3 condition); however, the maximum difference in the occlusion area was 19.84% (under the v3s3 condition). Under the s1 condition, the saccade speed of CI drivers in the occlusion area was lower than that of CNI drivers, but when the occlusion ratio was s2 and s3, the visual saccade speed of the CI drivers was higher than that of the CNI drivers. In addition, with the increase in the speed of visual stimuli, the differences in saccade speed of the two groups increased in the occlusion area.

A repeated measures 2 (driver group) × 3 (moving speed) × 3 (occlusion ratio) ANOVA was assessed for systematic trends, and a significant effect was found (F(2.514, 95.543) = 3.333, p = 0.030 < 0.05). ANOVA revealed a significant interaction of moving speed × occlusion ratio (F(2.566, 97.513) = 7.460, P < 0.001) and driver group × moving speed (F(1.508, 57.295) = 4.648, p < 0.05). No significant interactions were found for driver group × occlusion ratio (F(1.851, 70.335) = 4.797, P < 0.05). Greenhouse-Geisser corrections (ε) were made where assumptions of sphericity were violated.

Then, the interaction between the driver group and occlusion ratio on saccade speed was examined under different speeds of the visual stimulus. The result showed that there were no statistically significant interactions under the v1 (F(2, 76) = 2.579, P = 0.082 > 0.05) and v2 (F(2, 76) = 0.682, P = 0.509 > 0.05) conditions. A significant interaction (F(2, 76) = 4.642, P = 0.013 < 0.05) was found under the v3 condition.

Furthermore, the simple effect of the driver group was analyzed under the v3 condition. The results showed that there were no significant effects of the driver group on the saccade speed under the v3s1 (F(1, 38) = 0.157, P = 0.694 > 0.05) and v3s2 conditions (F(1, 38) = 3.759, P = 0.06 > 0.05). However, under the v3s3 condition, the saccade speeds of the CI and CNI driver groups were 9.107 ± 3.723°/s and 6.974 ± 2.077°/s, respectively. The difference between the two groups was statistically significant (F(1, 38) = 9.372, P = 0.004 < 0.05), with a difference of 2.133°/s (95% confidence interval: 0.723–3.544°/s). It is inferred that a high moving speed of the target and a longer occlusion area will cause a greater difference in the saccade speed. However, no significant differences were found in other states besides the v3s3 one.

#### 3.2.2 Recall times

Recall is a psychological prediction model of speed perception. When the speed of visual stimulation is high (v3), there are certain differences in visual perception patterns.

**Fig 7:**
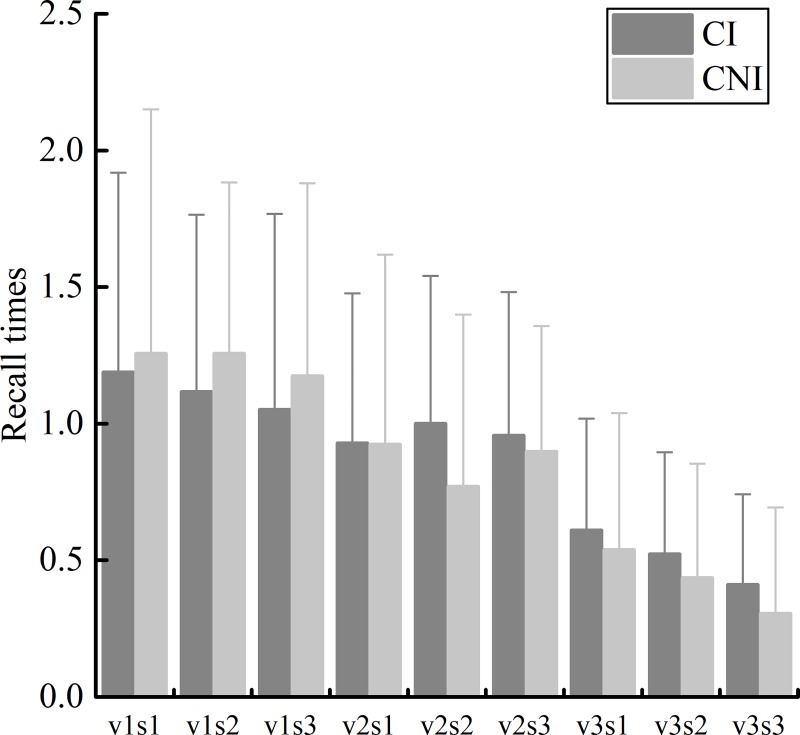


Subjects perceive the speed of visual stimuli by scanning the distance between the stimulus signal and the boundary and the time needed to reach the boundary. The angles of the boundary, differing in occlusion level, were approximately 3.37°, 6.74°, and 10.11°. The recall times of different subjects were measured (Fig 7). The independent sample t-test was used to analyze the differences between CI drivers and CNI drivers (Table 1).

**Table 1:** Result t-test of recall times of CI and CNI drivers in the visible area.

Status	CI		CNI		F	P—value
	Mean	SD	Mean	SD		
v1s1	1.19	0.73	1.26	0.89	0.202	0.806
v1s2	1.12	0.65	1.26	0.63	0.073	0.532
v1s3	1.05	0.71	1.17	0.72	0.184	0.623
v2s1	0.93	0.55	0.92	0.70	1.523	0.983
v2s2	1.00	0.54	0.77	0.63	0.940	0.255
v2s3	0.96	0.53	0.90	0.46	0.351	0.737
v3s1	0.61	0.41	0.54	0.50	0.520	0.652
v3s2	0.52	0.37	0.44	0.42	0.679	0.532
v3s3	0.30	0.33	0.41	0.39	1.391	0.393

The results showed that although there was a certain difference between the two groups in the mean values of the recall times, no significant difference was found under all conditions.

As shown in Fig 7, the recall times decrease with the moving speed of the visual stimulus and occlusion ratio for both CI and CNI drivers. When the speed of the visual stimulus was v1, the recall times of CNI drivers were more than those of CI drivers. However, contrary results were found when the target speed was v2 and v3.

#### 3.2.3 Visual pattern

**Fig 8:**
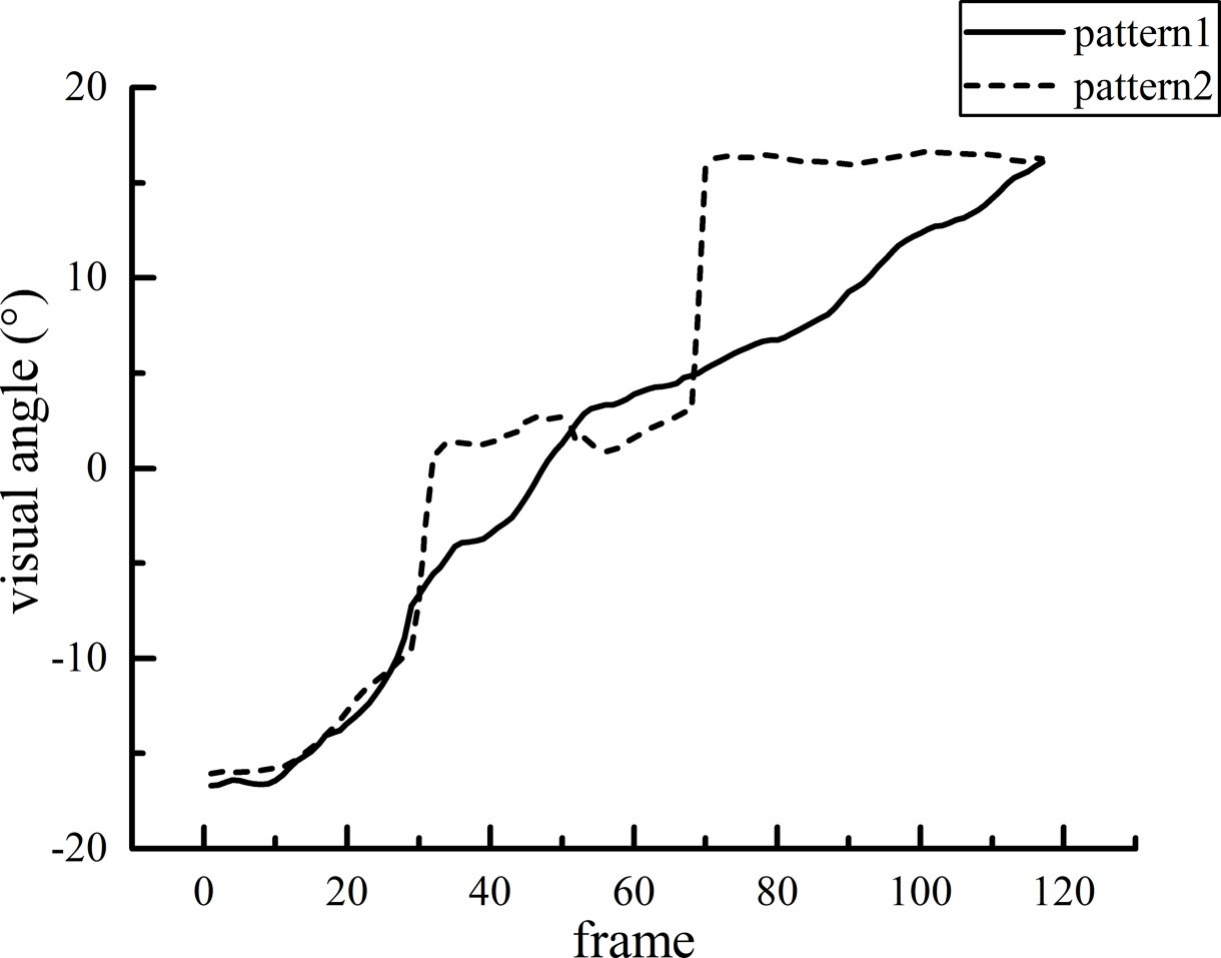


When the target moved at low or median speed, the subjects’ visual line could follow the target steadily and occasionally perform backward saccade (recall). Even in the occlusion area, the saccade speed was not much different from that in the visible area. However, when the target moved at high speed (v3), many differences in the saccade speed were found between the visible and occlusion areas. The eye movement of the subjects under the v3 situation in the occlusion area could be classified into the following two patterns (Fig 8).

The subjects visually simulated a virtual (invisible) stimulus signal and made a virtual smooth pursuit eye movement according to the perceived speed of the visual stimulus in the visible area. This process reflects the subjects' perception accuracy of visual stimulus signals and the accuracy of simulating these visual signals in visual psychology.

Fig 8 shows a diagram with an occlusion ratio of s1 (20%). The gaze dwells at the boundary of the visible and occlusion areas for approximately 40 frames at 3.5°/s.

**Fig 9:**
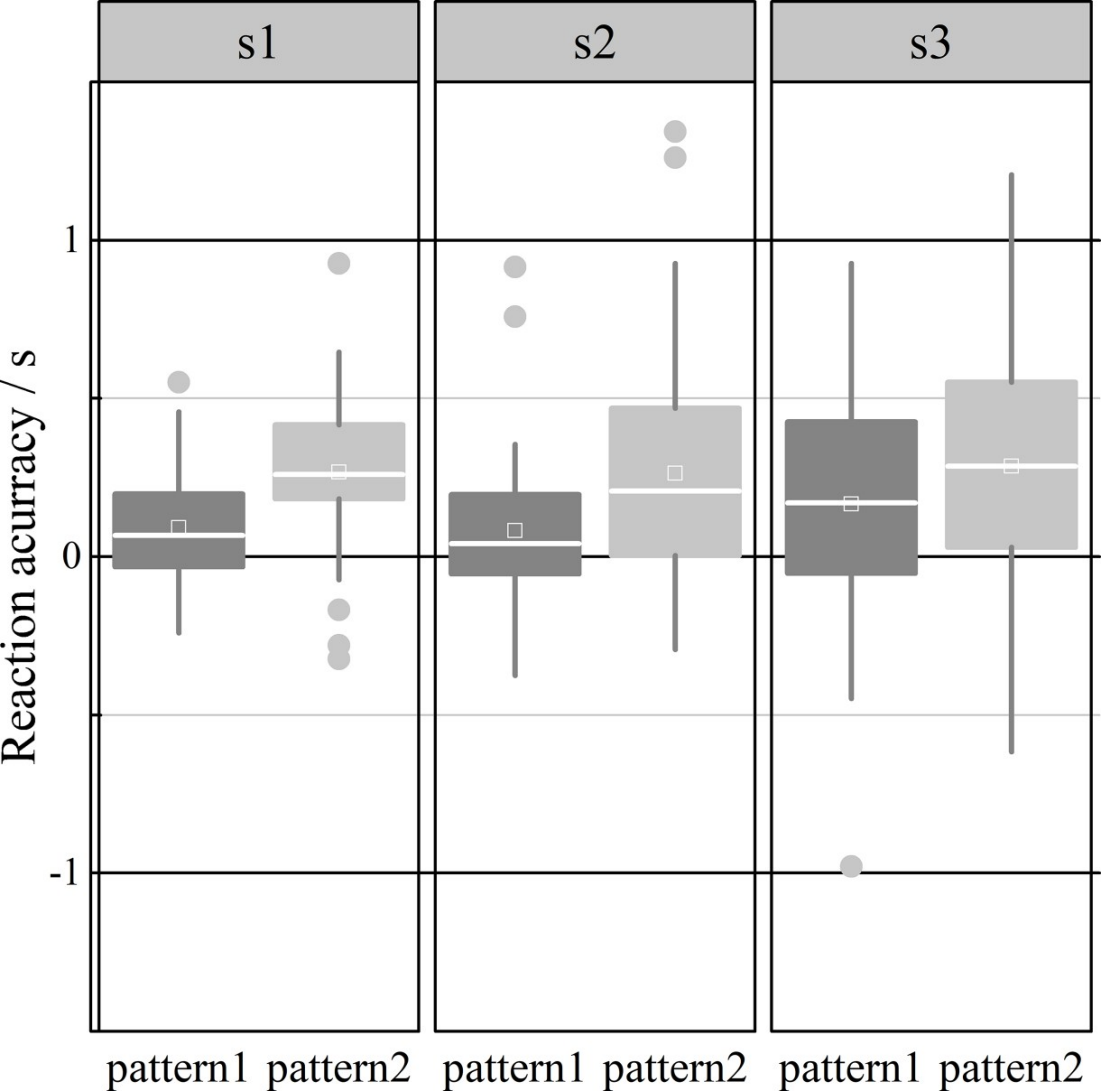


For pattern 1, the subjects imagine that there is an invisible virtual target moving in the occlusion area at the same speed as in the visible area. Therefore, they follow the virtual target with smooth pursuit until they make a response (press the button). For pattern 2, after the target enters the occlusion area, the line of sight dwells at the boundary of the visible and occlusion areas for a certain time and saccades to the end of the occlusion area. The participants estimate that the ball will react when it reaches the end point, which is characterized by obvious saccades in the occlusion area. The correlation between saccade pattern and reaction time is analyzed to reveal which saccade pattern is more accurate. Of course, each subject may have both of these visual patterns, and the occurrence is random. In this paper, it was assumed that there was a certain relationship between different visual patterns and the response accuracy. Therefore, the saccade patterns of all subjects were counted, and a t-test was conducted with the response accuracy. The response time accuracies of the subjects for different speed sensing patterns are shown in Fig 9.

As shown in Fig 9, speed perception was more accurate under pattern 1. In addition, more error was found with a higher occlusion percentage. It might be that the hypothetical target was more useful in movement perception. An independent sample t-test was used to verify the differences in reaction time error between the two patterns (Table 2).

**Table 2:** t-test results for different speed perception patterns.

Occlusion ratio	pattern 1		pattern 2		F	P—value
	Mean	SD	Mean	SD		
s1	0.09	0.17	0.27	0.26	2.36	0.000
s2	0.08	0.24	0.26	0.36	8.19	0.003
s3	0.17	0.38	0.29	0.39	0.41	0.115

Table 2 shows certain differences in the reaction accuracies for the two patterns. Significant differences in the reaction accuracy were found between the two patterns under s1 (F = 2.36, P < 0.001) and s2 (F = 8.19, P < 0.05). However, no significant difference was found under s3 (F = 0.41, P > 0.05). This result might have occurred because a higher occlusion area always resulted in larger reaction time errors.

## 4. Discussion

Our goal in this study was to identify differences in the speed perception abilities of crash-involved and crash-not-involved drivers and the eye movement characteristics in speed perception. In this paper, we proposed the concept of reaction in advance to characterize the impatience of drivers.

It is conceived that the slower the object speed and the longer the extrapolation interval, the greater the timing or localization error is [18]. As presented in Fig 5, the time accuracy decreased as the moving speed increased, which was consistent with the research result of Alexander [18, 19]. In addition, we found that the time accuracy was affected by the length of the visible path, which is consistent with the research result of Peterken et al. [30]. For instance, when the object moved at a slow speed (2.5°/s), the reaction error increased as the visible trajectory decreased (from 40% to 20%). It should be noted that significant effects of the visible trajectory percentage on time accuracy were found for both CI and CNI drivers.

Targets were perceived as moving faster in trials containing forward (catch-up) saccades compared with trials containing backward saccades. Targets tracked with a pure pursuit response were perceived to move at a close-to-veridical intermediate speed [1]. In our study, we defined the recall process to describe the participant’s forward saccade to the boundary of the visible and occlusion areas and then backward saccade to the visual target. Therefore, both forward and backward saccades were contained in the recall process. However, there were no significant differences in recall times between CI and CNI drivers. It might be possible that in the visible area, the pursuit pattern has no significant impact on the accuracy of the perceived speed of the target. Based on the eye movement data, we defined two kinds of visual patterns in our paper when the moving speed of the target was 10°/s. The literature review found that no similar study methods had been proposed before. According to the statistics, we found significant differences in time accuracy when the occlusion ratios were s1 and s2. Based on the results, we believe that visual patterns have an important impact on speed perception accuracy. However, no significant difference was found when the occlusion ratio was s3. This result might reflect the fact that the uncertainty increased as the occlusion trajectory became longer. Therefore, we concluded that visual patterns might be useful to some extent.

These findings suggest that speed perception may be a useful method to distinguish drivers who are prone to being involved in rear-end or side collision traffic accidents. It is suggested that speed perception is available for driver aptitude tests. However, the results of this study might have been different (more or less significant) if the sample size was fully balanced in terms of gender and driving experience. Further studies are needed to investigate whether gender and driving experience affect speed perception accuracy.

## Supporting information

S1 Data (TXT)
